# Towards an emotional ‘stress test’: a reliable, non-subjective cognitive measure of anxious responding

**DOI:** 10.1038/srep40094

**Published:** 2017-01-10

**Authors:** Jessica Aylward, Oliver J. Robinson

**Affiliations:** 1 Institute of Cognitive Neuroscience, UCL, 17–19 Queen Square, London, WC1N 3AZ, United Kingdom

## Abstract

Response to stress or external threats is a key factor in mood and anxiety disorder aetiology. Current measures of anxious responding to threats are limited because they largely rely on retrospective self-report. Objectively quantifying individual differences in threat response would be a valuable step towards improving our understanding of anxiety disorder vulnerability. Our goal is to therefore develop a reliable, objective, within-subject ‘stress-test’ of anxious responding. To this end, we examined threat-potentiated performance on an inhibitory control task from baseline to 2–4 weeks (n = 50) and again after 5–9 months (n = 22). We also describe single session data for a larger sample (n = 157) to provide better population-level estimates of task performance variance. Replicating previous findings, threat of shock improved distractor accuracy and slowed target reaction time on our task. Critically, both within-subject self-report measures of anxiety (ICC = 0.66) and threat-potentiated task performance (ICC = 0.58) showed clinically useful test-retest reliability. Threat-potentiated task performance may therefore hold promise as a non-subjective measure of individual anxious responding.

Mood disorders are common, but there is huge variability in vulnerability^1^. According to the diathesis–stress model^2^, a disorder is triggered when an underlying vulnerability, coupled with stressful life events reaches a threshold. This suggests that a clear ability to quantify mood and anxiety disorder vulnerability will not emerge without an ability to quantify individual differences in anxious responding or sensitivity to threat.

Subjective ratings of self-reported anxiety levels can be increased experimentally using threat of unpredictable shock. In this paradigm, unpredictable electric shocks are delivered to the wrist, independent of task performance. This manipulation engages similar circuitry as pathological anxiety^3^. ‘Threat-potentiated’ responding can then be determined by comparing performance in the same individual when they are at risk and safe from shock. Many domains of cognition are affected (for a review see ref. 4) including inhibitory control^5^ and attentional bias towards threat^6^.

The Sustained Attention to Response Task (SART), where participants respond to frequent target stimuli whilst withholding a response to infrequent distractor stimuli (under alternating threat and safe conditions where at risk and safe from shock, respectively), can explore the interaction between threat-potentiated responding and inhibitory control. There is strong evidence to support the hypothesis that the impact of induced anxiety on this task (which can be maladaptive or adaptive depending on the context) is a consequence of facilitated motor response inhibition^5^. The impact of threat of shock on accuracy has been confirmed in numerous studies, improving accuracy to distractor stimuli^5^ and impairing accuracy to target stimuli^7^, but its test-retest reliability is unknown. The impact on reaction time is less clear; in some cases there are no effects^5^,^8^ whilst in a modified version of the SART an increase in reaction time was evidenced (see preprint and data doi:10.7287/PEERJ.PREPRINTS.1542V1).

In the cardiac ‘stress-test’, subjecting the heart to the stress of exercise reveals key diagnostic signatures of heart disease vulnerability that are not evident at rest^9^. Here we seek to develop an emotional analogue of this ‘stress test’. In order to have clinical value, test performance needs to meet certain assumptions, for example; good validity, the degree to which a test accurately measures what it is intended to measure, and (as we are probing here), it should be reliable and stable across time in the same individuals^10^. Many cognitive tasks, nevertheless, show poor reliability (see Table 1).

We therefore probed the test-retest reliability of threat-potentiated responding on the SART using Intraclass Correlation Coefficients^11^. We predicted that threat of shock would improve accuracy at withholding a response to distractor stimuli, in line with previous findings. Critically, we predicted that this would be reliable across testing sessions and hence constitute a non-subjective, behavioural measure of individual anxious responding: an emotional equivalent of the cardiac ‘stress test’. In order to provide a more accurate estimation of the population level mean and variance – critical prior to any clinical application – we also analysed data from a larger, heterogeneous sample (N = 157).

## Results

Test-retest reliability analyses were run on the individual measures across two and three testing sessions (See Tables 2, 3 and 4, respectively).

### Accuracy to “no go” stimuli

**Figure 1:**
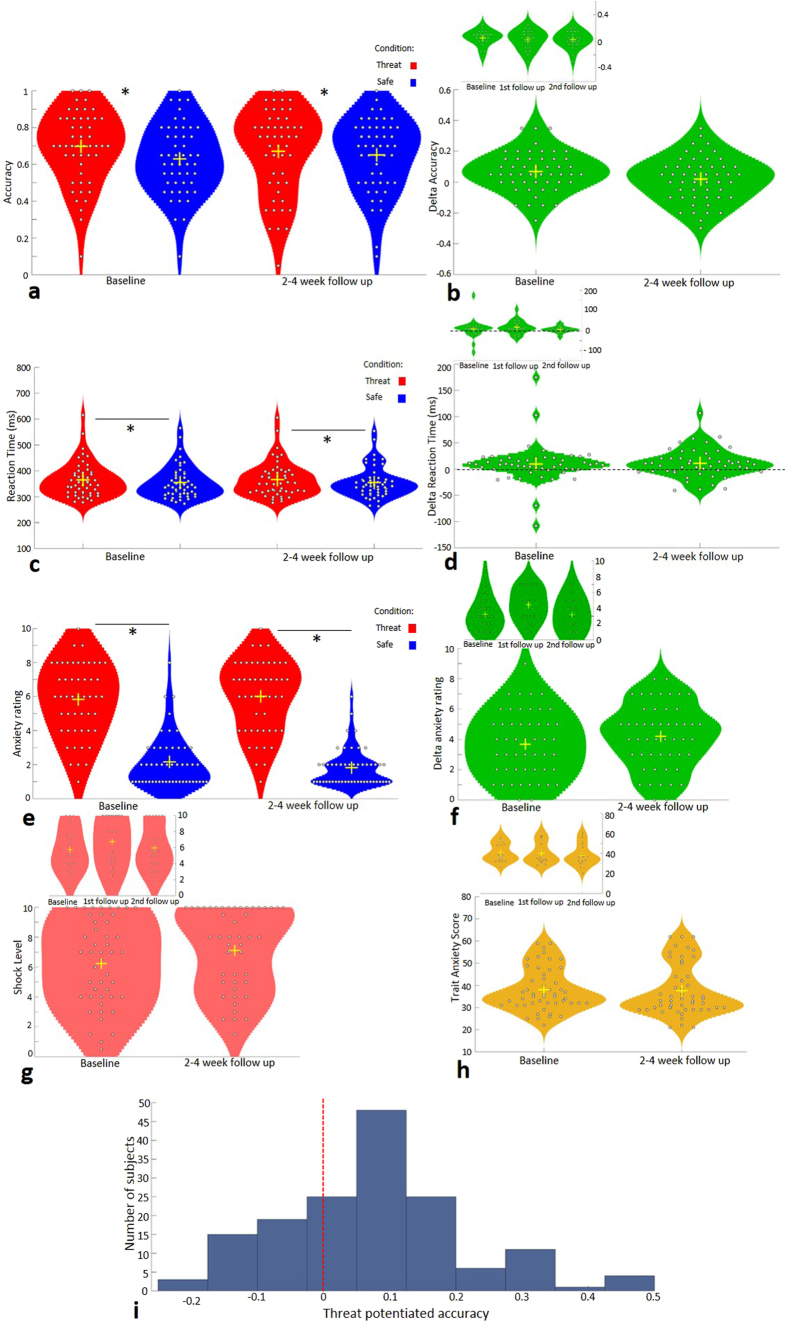
Violin plots (shaded area represents a histogram) (**a**). Accuracy to “no go” stimuli across threat and safe conditions. There was a main effect of condition (*p* = 0.04). (**b**) Delta accuracy (inset across three sessions). (**c**) Reaction time to “go” stimuli across threat and safe conditions. There was a main effect of condition (*p* = 0.012). (**d**) Delta reaction time (inset across 3 sessions). (**e**) Anxiety rating across threat and safe conditions. There was a main effect of condition (*p* < 0.05). (**f**) Delta anxiety ratings (inset across three conditions). (**g**) Shock level across baseline and follow up (main effect of session *p* = 0.003; inset shock level across three sessions). (**h**) Trait anxiety score across testing sessions (inset trait anxiety across three sessions). (**i**) Distribution of delta distractor accuracy scores on the SART in a large population (N = 157). Dotted line at zero demonstrates population as a whole shifted towards threat-potentiated accuracy.

A repeated measures ANOVA including condition and session revealed a main effect of condition (F_(1,49)_ = 9.11, *p* = 0.004, 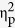 = 0.157) (Fig. 1a,b). Participants were significantly more accurate under threat of shock (threat mean = 0.685, SD = 0.20; safe mean = 0.641, SD = 0.18). There was no main effect of session or a significant threat x session interaction (*p* = 0.93, *p* = 0.063, respectively). The reliability of threat induced accuracy changes across 2 sessions had a non-significant ICC of 0.23 (F_(49,49)_ = 1.31, *p* = 0.17, 95% CI −0.32, 0.55) but across 3 sessions, the effect of threat on accuracy was significant and “fair to good”, with an ICC of 0.51 (F_(21,42)_ = 2.02, *p* = 0.026, 95% CI −0.0026, 0.78).

For those participants who completed two sessions only, the reliability of threat induced accuracy had a non-significant ICC of 0.31 (F_(27,27)_ = 1.49, *p* = 0.15, 95% CI −0.38, 0.67).

### Reaction time to “go” stimuli

A repeated measures ANOVA including condition and session revealed a significant effect of condition (F_(1,49)_ = 6.75, *p* = 0.012, 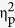 = 0.121) (Fig. 1c,d). Participants were slower to respond during the threat condition relative to the safe condition (threat mean = 365.40, SD = 64.71; safe mean = 355.44, SD = 56.73). There was no effect of session, nor a session x condition interaction (*ps* > 0.250). Reliability for the effect of threat of shock across 2 sessions was significant and “fair to good” with an ICC of 0.58 (F_(49,49)_ = 2.38, *p* = 0.0015, 95% CI 0.26, 0.76) and remained “fair to good” across 3 sessions with an ICC of 0.50 (F_(21,42)_ = 1.99, *p* = 0.029, 95% CI −0.017, 0.78).

For those participants who completed two sessions only, the effect of induced anxiety on reaction time was non-significant, with an ICC of 0.42 (F_(27,27)_ = 1.73, *p* = 0.08, 95% CI −0.25, 0.73).

### Anxiety Rating

Participants were more anxious during the threat condition relative to the safe condition (F_(1,49)_ = 225.96, *p* < 0.001, 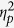 = 0.82)(Fig. 1e,f). There was no main effect of session or a significant session x condition interaction (*p* = 0.75, *p* = 0.089, respectively). The reliability of this effect was “fair to good” across 2 sessions with an ICC of 0.66 (F_(49,49)_ = 3.05, *p* < 0.001, 95% CI 0.41, 0.81) and “excellent” across 3 sessions with an ICC of 0.75 (F_(20,40)_ = 3.90, *p* < 0.001, 95% CI 0.48, 0.89).

For those participants who completed two sessions only, the reliability of this effect was “excellent” with an ICC of 0.77 (F_(27,27)_ = 4.20, *p* < 0.001, 95% CI 0.49, 0.89).

### Shock Level

Shock level was significantly higher in the second session (F_(1,49)_ = 9.62, *p* = 0.003, 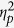 = 0.164; session 1 mean = 6.22, SD = 2.89; session 2 mean = 7.10, SD = 2.79)(Fig. 1g).

Reliability for shock level over 2 sessions was significant, with an “excellent”, with an ICC of 0.84 (F_(49,49)_ = 7.05, *p* < 0.001, 95% CI 0.68, 0.91) and reliable across 3 sessions with an “excellent” ICC of 0.93 (F_(21,42)_ = 16.20, *p* < 0.001, 95% CI, 0.85, 0.97).

For those participants who completed two sessions only, the reliability for shock level was “excellent” with an ICC of 0.80 (F_(27,27)_ = 5.24, *p* < 0.001, 95% CI 0.56, 0.91).

### Trait Anxiety

Trait anxiety scores were also analysed as a comparison. There was no significant change over session (*p* = 0.622) (Fig. 1h). The reliability of the trait anxiety score was “excellent”, with an ICC of 0.95 across 2 sessions (F_(49,49)_ = 20.97, *p* < 0.001, 95% CI 0.92, 0.97) and across 3 sessions with an “excellent” ICC of 0.94 (F_(21,42)_ = 16.67, *p* < 0.001, 95% CI, 0.87, 0.97).

For those participants who completed two sessions only, the reliability for trait anxiety was significant, with an “excellent” ICC of 0.96 across 2 sessions (F_(27,27)_ = 26.40, *p* < 0.001, 95% CI 0.92, 0.98).

### Population variance

Threat potentiated accuracy to no-go stimuli (threat minus safe accuracy at correctly withholding a response to distractor stimuli), in 157 subjects revealed increased a population mean of 0.072, median of 0.05, a standard deviation of 0.15 (See Fig. 1).

## Discussion

We show that threat of shock can reliably shift within-subject cognitive and self-report measures of anxious responding across three sessions and three quarters of a year, improving accuracy to distractor stimuli and slowing down responses to target stimuli. This improvement in accuracy replicates previous studies^5^,^8^ and an increase in reaction time under threat of shock replicates a line of previous research (see preprint and data*).

Importantly, threat-potentiated performance on this task also shows good within-subject reliability over 2–4 weeks in the full sample and again over a 5–9 months. For reference, this means that the emotional manipulation (i.e. threat of shock) on this task, is considerably more reliable than the emotional manipulation (i.e. emotional stimuli) on the emotional Stroop and dot probe tasks^12^,^13^(see Table 1).

We note that there is imprecision surrounding the term, ‘stress’, which is used to describe the prolonged HPA axis response or psychological experiences (of which there is a perception of a mechanistic link^14^,^15^). Considering the time course of the glucocorticoid response and our manipulation, it is unlikely this link would be observed using the present design. We regard our findings as indicative of anxious responding and use the term, ‘stress test’, because our goals align with those of the cardiac ‘stress test’. ‘Stress tests’ are of great value in cardiac medicine as they are able to identify patients who may be more vulnerable and need closer monitoring around surgery, which in turn leads to improved outcomes^16^.

Prior work has shown that participants with clinical anxiety showed impaired “go” accuracy overall compared to controls on this, suggesting that there is an overactive adaptive defensive mechanism in clinical anxiety^17^. Consequently, we suggest that the effect of threat on reaction time observed here could be a reflection of this behavioural inhibition. The impact of induced anxiety on response inhibition could therefore be a behavioural marker for clinical anxiety so, given the reliability of this task, we believe is has clinical potential.

According to the diathesis model of anxiety disorders, when stressful life events are coupled with an underlying vulnerability and a threshold is reached, a disorder is triggered. Understanding individual responses to stress is therefore key to understanding the differences in vulnerability to anxiety disorders. Pathological feelings of anxiety contribute to the most common psychiatric disorders, and it is suggested that over the next 20 years these rates will continue to rise^18^. Identifying vulnerability prior to disorder onset with a non-subjective cognitive task could consequently lower costs and reduce time in treatment. Additionally, cognitive paradigms which show good reliability are important for research, impacting replicability and the accurate interpretation of existing findings^19^.

It should be noted that self-report trait anxiety also has a high ICC in this study. However, interpretation of this is limited due to anchoring effects^20^ and demand characteristics^21^. Our task is not obviously subject to these effects and also benefits from being a concurrent (i.e. not retrospective) measure. The poorer test-retest reliability on our accuracy delta variable for the two to four week follow up may be due to a reduction in power resulting from the smaller number of no-go responses, and suggests that go reaction time differences may prove the more reliable target. It is also worth noting that we see poorer test-retest reliability in the sub-sample who only completed two sessions (Table 3). The reasons for this are unclear, but may be driven by self-selection bias in individuals unwilling or unable to return for a third testing session. Of course, this sample is also underpowered per our power calculation so inference should be approached with caution.

In summary, we argue that the impact of threat of shock on cognition might hold promise as a putative probe of threat sensitivity, and a phenotype of anxious responding.

## Method

Fifty healthy participants (25 female, mean age = 26.5, SD = 8.47), completed the SART in two testing sessions, separated by a period of between two and four weeks. Twenty two participants (11 female, mean age = 28.5, SD = 11.00) completed the task for the third time in a follow up session between five and nine months later. A screening procedure prior to participation verified that participants had no history of neurological, psychiatric, or cardiovascular conditions. Exclusion criteria also included alcohol dependence and any recreational drug use in the last 4 weeks.

The methods were identical on each session. Participants provided written informed consent to take part in the study (UCL ethics reference: 1764/001). Prior to participation, subjects were screened to ensure that they had no history of neurological, psychiatric, or cardiovascular conditions. All methods were carried out in accordance with relevant guidelines and regulations and all protocols were approved by UCL ethics committee (reference 1764/001).

An *a priori* power analysis was run in G*Power^22^. The power analysis was based on previous results of the SART^5^ that gave an effect size of 0.56 for the effect of threat of shock on response accuracy to “no-go” distractor stimuli. We wanted 95% power (with alpha 0.05, two tailed) to detect an effect size of 0.56. A power calculation determined that we needed 46 participants. We recruited an extra 4 to allow for ~8% participant drop-off. This sample size also has 99% power to detect a reliability of at least 0.5 (a minimum value we consider acceptable for clinical relevance) at alpha = 0.05 (one-tailed). For the final 5–9 month follow up we showed considerable (56%) drop-off. A post hoc matched t-test power analysis showed that with 22 participants (with alpha = 0.05, two tailed) we had only 70.96% power to detect an effect of this magnitude. Notably, however, this still has 83% power to detect a reliability of at least 0.5 (a minimum value we consider acceptable for clinical relevance) at alpha = 0.05 (one-tailed). As such, this three-session analysis is powered for reliability analysis only. Given that 3 session and full 2 session samples overlap, we also include a separate 2 session analysis on those who only attended twice for completeness.

### Anxiety manipulation

Two electrodes were attached to the back of the participants’ non-dominant wrist. A Digitimer DS5 Constant Current Stimulator (Digitimer Ltd., Welwyn Garden City, UK) delivered the shocks. A short shock-level work up increased the level of the shock until the subject rated it as “unpleasant, but not painful”^23^. As in previous versions of this task^5^ during a threat block, in which the background was red, the participants were told they were at risk of an unpredictable shock (which was independent of their behavioural response). When in a safe block, the background was blue (and participants were told that no shocks would be delivered). Colours were not counterbalanced as prior work has shown this effect to be independent of background colour^24^,^25^. After completing the task, participants provided retrospective subjective anxiety ratings for the threat and safe conditions. This manipulation check is consistent with current clinical diagnoses (i.e. self-report of symptoms) and is used by many other researchers in the field (for a review see ref. 4).

### Task structure

Participants completed a previously used task^5^ recoded using the Cogent (Wellcome Trust Centre for Neuroimaging and Institute of Cognitive Neuroscience, UCL, London, UK) toolbox for Matlab (2014b, The MathWorks, Inc., Natick, MA, United States). Participants were instructed to respond to “go” target stimuli (“=”) by pressing the space bar as quickly as possible, and withhold a response to “no go” target stimuli (“O”). They were instructed to make their response using their dominant hand.

**Figure 2:**
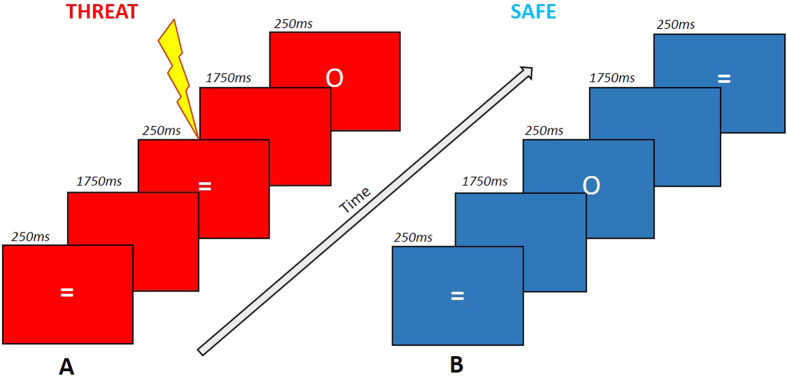
Participants were instructed to press the space bar as quickly as possible for “go” stimuli and withhold responses to infrequent “no-go” stimuli. (**A**) Participants received an unpredictable electric shock (independent of behavioural response) during the threat condition. (**B**) Participants were not at risk of shock during the safe condition.

47 “go” stimuli and 5 “no-go” stimuli were presented in each block. The stimuli were presented for 250 ms, followed by an interstimulus interval of 1750 ms, before presentation of the next stimulus. There were 8 blocks in total, alternating between threat and safe blocks (order counterbalanced). Each block lasted 104 seconds (See Fig. 2). For 3 seconds at the beginning of each block, “YOU ARE NOW SAFE FROM SHOCK!” or “YOU ARE NOW AT RISK OF SHOCK!” appeared on the screen. Participants received a shock in the first threat block, (after trial 45), the second threat block (after trial 8), and the fourth threat block (after trial 17). Total task duration was approximately 14 minutes and 30 seconds (task script available online: https://figshare.com/articles/SART_script/3443093).

### Wider sample

Threat-potentiated task performance data from a larger (n = 157) heterogeneous sample collected across UCL, UK and NIH, USA are also presented to explore population level statistics.

### Statistical Analyses

Reaction time and ac**c**uracy data (data available online: https://dx.doi.org/10.6084/m9.figshare.3398764.v1) were analysed using repeated-measures general linear models in SPSS version 22 (IBM Crop, Armonk, NY). For all analyses, *p* = 0.05, was considered significant. Performance accuracy for each condition (threat/safe) and trial type (“go”/“no-go”) was calculated by dividing the number of correct trials by the total number of trials. As “go” accuracy was 97.5% across two sessions, only “no-go” trials were included in the accuracy analysis. Reaction time analysis was performed on “go” stimuli only as, by definition, “no-go” reaction times are limited and restricted to error trials.

For the first two fully powered sessions, repeated measures ANOVAs were run to investigate reaction time and accuracy differences across conditions. Due to lack of power resulting from attrition (see above) these were not run for the third session.

Task reliability over two and three sessions was tested using two-way mixed model ICCs run in Matlab (2014b) using an “Intraclass Correlation Coefficient” script (http://uk.mathworks.com/matlabcentral/fileexchange/22099-intraclass-correlation-coefficient—icc-). This determined whether the influence of threat of shock on various performance measures remained consistent in individuals between testing sessions. In accordance with^11^ an ICC coefficient was considered ‘fair to good’ if between 0.4 and 0.75, and ‘excellent’ if above 0.75. In our power calculation we deemed 0.5 the minimum reliability required for clinical relevance. Reliability analyses were completed on the critical delta variables (the difference between threat and safe condition for that variable) to look at the reliability of the *threat-potentiated* effect for reaction time, accuracy and shock rating. Analyses were also run for shock level and trait anxiety scores (for which there are only one measurement per session, so no deltas). Estimates for each condition separately demonstrating the reliability of the individual measures themselves are presented in Table 2, 3 and 4.

## Additional Information

**How to cite this article**: Aylward, J. and Robinson, O. J. Towards an emotional ‘stress test’: a reliable, non-subjective cognitive measure of anxious responding. *Sci. Rep.* **7**, 40094; doi: 10.1038/srep40094 (2017).

**Publisher's note:** Springer Nature remains neutral with regard to jurisdictional claims in published maps and institutional affiliations.

## Figures and Tables

**Table 1:** Commonly used emotional tasks and their test-retest reliabilities.

Emotional Task	Test-retest reliability	Type of reliability measure	Reference
Emotional Stroop	−0.17	(Anxiety - Neutral) ‘Reliability coefficient’	26
	0.29	(Standard Stroop interference) Pearson’s *r*	12
Dot probe	−0.04	(Self-relevant positive words) ‘Reliability coefficient’	27
	0.04	(Social threat words) ‘Reliability coefficient’	28
	0.13	(Negative unmasked) Two way mixed ICC	13

Note that for the reliability coefficients/Pearsons’s r: 0.7 is strong, 0.5 is moderate and 0.3 is weak reliability. For the ICCs, 0.4–0.75 is ‘fair to good’ reliability and >0.75 is ‘excellent’ reliability.

**Table 2:** Full sample reliability of measures across two testing sessions (N = 50).

Individual measures of interest	ICC value	Repeated measures ANOVA
Accuracy to “no-go” stimuli across safe conditions	0.82*	F_(49,49)_ = 5.71
Accuracy to “no-go” stimuli across threat conditions	0.87*	F_(49,49)_ = 7.58
Difference in accuracy to “no-go” stimuli across conditions	0.23	F_(49,49)_ = 1.31
Reaction time to “go” stimuli across safe conditions	0.82*	F_(49,49)_ = 5.40
Reaction time to “go” stimuli across threat conditions	0.91*	F_(49,49)_ = 11.14
Difference in reaction time to “go” stimuli across conditions.	0.58*	F_(49,49)_ = 2.37
Self-report anxiety level across safe conditions	0.63*	F_(49,49)_ = 2.78
Self-report anxiety level across threat conditions	0.62*	F_(49,49)_ = 2.59
Difference in self-report anxiety level across conditions	0.66*	F_(49,49)_ = 3.05

^*^p < 0.05.

**Table 3:** Reliability of measures across participants who completed two testing sessions only (N = 28).

Individual measures of interest	ICC value	Repeated measures ANOVA
Accuracy to “no-go” stimuli across safe conditions	0.86*	F_(27,27)_ = 7.39
Accuracy to “no-go” stimuli across threat conditions	0.85*	F_(27,27)_ = 6.85
Difference in accuracy to “no-go” stimuli across conditions	0.31	F_(27,27)_ = 1.31
Reaction time to “go” stimuli across safe conditions	0.68*	F_(27,27)_ = 3.21
Reaction time to “go” stimuli across threat conditions	0.82*	F_(27,27)_ = 5.61
Difference in reaction time to “go” stimuli across conditions.	0.42	F_(27,27)_ = 1.73
Self-report anxiety level across safe conditions	0.63*	F_(27,27)_ = 2.73
Self-report anxiety level across threat conditions	0.75*	F_(27,27)_ = 3.94
Difference in self-report anxiety level across conditions	0.77*	F_(27,27)_ = 4.20

^*^p < 0.05.

**Table 4:** Reliability of measures across participants who completed three testing sessions (N = 22).

Individual measures of interest	ICC value	Repeated measures ANOVA
Accuracy to “no-go” stimuli across safe conditions	0.80*	F_(21,42)_ = 4.94
Accuracy to “no-go” stimuli across threat conditions	0.91*	F_(21,42)_ = 11.87
Difference in accuracy to “no-go” stimuli across conditions	0.51*	F_(21,42)_ = 2.02
Reaction time to “go” stimuli across safe conditions.	0.88*	F_(21,42)_ = 9.21
Reaction time to “go” stimuli across threat conditions.	0.93*	F_(21,42)_ = 16.53
Difference in reaction time to “go” stimuli across conditions.	0.50*	F_(21,42)_ = 1.99
Self-report anxiety level across safe conditions	0.83*	F_(20,40)_ = 6.84
Self-report anxiety level across threat conditions	0.60*	F_(20,40)_ = 2.58
Difference in self-report anxiety level across conditions	0.75*	F_(20,40)_ = 3.90

^*^p < 0.05.
